# Intrauterine device found in an ovarian tumor: A case report

**DOI:** 10.1097/MD.0000000000022825

**Published:** 2020-10-16

**Authors:** Yongyu An, Chang Liu, Fan Mao, Guangzhao Yang, Guoqun Mao

**Affiliations:** Department of Radiology, Tongde Hospital of Zhejiang Province, Hangzhou, Zhejiang Province, China.

**Keywords:** intrauterine device, ovarian tumor, uterine perforation

## Abstract

**Rationale:**

Intrauterine devices (IUDs) are one of the most common and effective methods of contraception worldwide. Migration of an IUD to an extrauterine site is a rare complication. The aim of this study was to report an extremely rare case in which an IUD was found in an ovarian tumor.

**Patient concerns:**

A 63-year-old Chinese woman presented with vaginal bleeding and lower abdominal pain during hospitalization due to pneumonia. Preoperative imaging showed bilateral cystic masses in the adnexal region, and ring hyperdensity was found in the right ovarian mass. Endometrial thickening and multiple uterine leiomyomas were found on ultrasonography. Hysteroscopy showed partial septate uterus and a small endometrial polyp.

**Diagnosis:**

Bilateral ovarian cystadenomas with perforation of the IUD into the right ovarian tumor were considered based on preoperative imaging and the patient's medical history. Furthermore, early endometrial carcinoma was suspected.

**Interventions:**

The patient underwent hysterectomy, bilateral salpingo-oophorectomy, and omentectomy. A stainless steel ring IUD was confirmed within the right ovarian tumor during the operation.

**Outcomes:**

The pathology results demonstrated bilateral ovarian serous cystadenofibromas with focal epithelial proliferation and endometrial atypical hyperplasia with malignant transformation. The patient has been followed up for 7 months, and there has been no recurrence at present.

**Lessons:**

The presence of an IUD within an ovarian tumor is extremely rare. This is the second reported case in the English literature describing an extrauterine IUD within an ovarian tumor. The correlation between ovarian cancer tumorigenesis and IUD translocation is unclear and requires further investigation.

## 1 Introduction

Intrauterine devices (IUDs) are a widely used method for contraception worldwide. The utilization rate of IUDs in the USA and Europe are approximately 2% and 6%, respectively.^[1,2]^ In China, IUD insertions are more prevalent, and approximately 44% of women between 15 and 45 years of age use IUDs because of the 1-child policy.^[3]^ Nevertheless, IUDs are not risk free, and IUD migration is a frequently encountered complication in clinical practice, varying from uterine expulsion to uterine perforation. Partial or complete perforation of the IUD is a rare but serious complication. The incidence rate of uterine perforation is 0.3 to 2.6 in every 1000 copper IUD insertions and 0.3 to 2.2 for levonorgestrel-releasing intrauterine system insertions.^[4–7]^ An ectopic IUD may adhere to the omentum or adjacent bowel or penetrate into the adjacent viscera. There are several cases of transmigrated IUDs in the extrauterine viscera that have been reported in the literature, such as in the bladder, rectum, and appendix.^[8–10]^ Migration of the IUD into the ovary is rare, especially into ovarian tumors, although the ovary is proximal to the uterus. We herein report an extremely rare case of IUD migration into an ovarian tumor.

## 2 Case presentation

A 63-year-old Chinese woman presented to the Department of Respiration for persistent cough for 3 months, and she was admitted for pneumonia. During hospitalization, she complained of vaginal bleeding and lower abdominal pain. She denied any history of vaginal bleeding after menopause. Physical examination identified a large hard mass in the abdomen and pelvis. Routine serum tumor marker examination showed elevated levels of CA125 and CA199 (158.10 U/mL, 251.96 U/mL, respectively). The C-reactive protein level was 56.6 mg/L. The fasting blood glucose and 2-hour post load glucose levels were 9.07 mmol/L and 14.45 mmol/L, respectively. The glycated hemoglobin A1c level was 10.0%. The test results for blood count, liver and renal function, hepatitis were negative.

**Figure 1:**
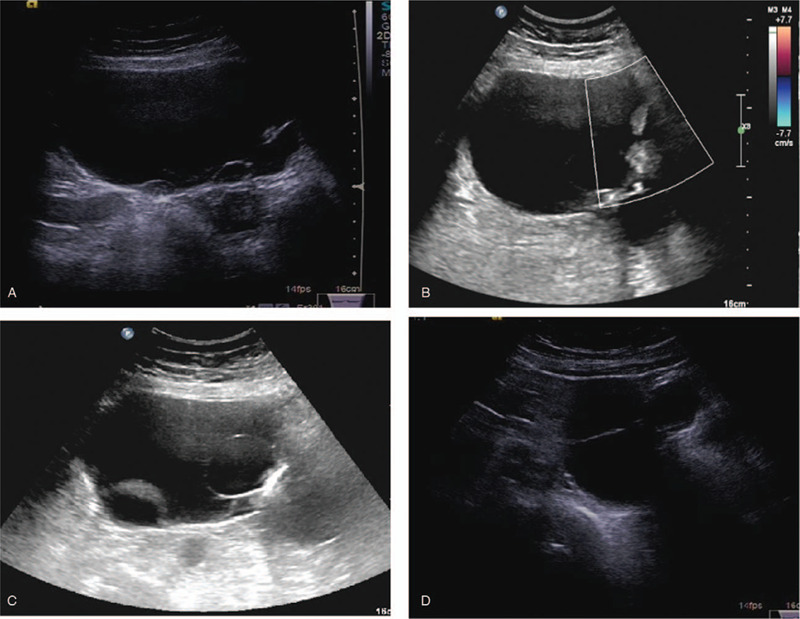
(A) Transabdominal US showed a large multiloculated cystic mass in the right adnexa. (B) No blood flow signals were detected in the papillary projection of the mass by Doppler US. (C) A liner high echo signal with shadows was found within the mass. (D) Another multiloculated mass with thin septa was also seen in the left adnexa. US = ultrasonography.

**Figure 2:**
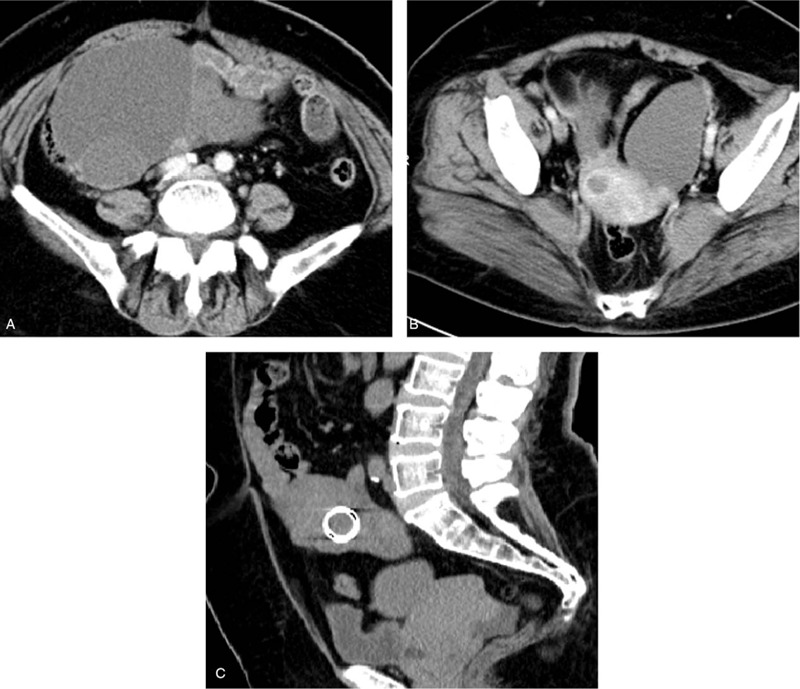
(A) Axial contrast-enhanced CT images showed a cystic mass with enhanced small nodules in the right adnexa. (B) Another cystic mass was also found in the left adnexa. (C) Coronal multiplanar reconstruction confirmed ring hyperdensity within the right ovarian mass, implying migration of the IUD. CT = computed tomography, IUD = intrauterine device.

Transabdominal ultrasonography (US) demonstrated a large multiloculated cystic mass (15 × 12 cm) with thin septa and multiple papillary projections in the right adnexa (Fig. 1A and B), and a linear structure with shadows was found within the mass (Fig. 1C). Another similar multiloculated cystic mass (8 × 7 cm) with thin septa was also detected in the left adnexa (Fig. 1D). Moreover, endometrial thickening (11 mm) with heterogeneous echogenicity and multiple uterine leiomyomas (maximum diameters: 3.3 cm) were found on US. Computed tomography (CT) imaging showed bilateral cystic ovarian masses, with multiple enhanced small nodules in the right mass (Fig. 2A and B). Ring hyperdensity was found in the right ovarian mass (Fig. 2C), which indicated malposition of the IUD. No peritoneal thickening or nodules were observed. There was a small amount of fluid collection in the Douglas pouch. Additional obstetric history was required. The patient remembered that she had first delivery 37 years ago and then had an IUD insertion one and a half years later. While still retaining the IUD, she became pregnant 3 months later. She chose to receive an abortion, but she was uncertain if the IUD was taken out at the same time. Considering the imaging features and the patient's medical history, bilateral ovarian cystadenomas with perforation of the IUD into the right ovarian tumor were diagnosed. Furthermore, early endometrial cancer was suspected. Hysteroscopy showed partial septate uterus and a small endometrial polyp. Surgery was suggested in view of the preoperative examination and clinical symptoms.

Laparotomy was performed. During the operation, bilateral ovaries were abundant in serous fluid, with multiple papillary projections into the lumen. A stainless steel ring IUD was positioned in the right ovarian mass. There was no evidence of extraovarian nodules except on the surface of the left fallopian tube. Intraoperative frozen sections revealed bilateral ovarian serous tumors with borderline portions. The patient underwent hysterectomy, bilateral salpingo-oophorectomy, and omentectomy. The final pathological findings were bilateral ovarian serous cystadenofibromas with focal epithelial proliferation and endometrial atypical hyperplasia with malignant transformation. Malignancy was not discovered in the omentum and peritoneum. The postoperative course was uneventful, and the patient was discharged several days later. No recurrence was found after a follow-up of 7 months.

## 3 Discussion

Complications associated with IUDs are not uncommon in clinical practice, although IUDs are highly effective and safe. The major complications include expulsion, displacement, pelvic inflammatory disease, uterine perforation, and ectopic pregnancy.^[11]^ The most serious, but rare complication is uterine perforation, which may cause serious symptoms. Perforation occurs most frequently at the time of insertion due to immediate traumatic perforation.^[6,12]^ Some perforations may occur later due to gradual erosion of the uterine wall.^[13]^ Moreover, uterine contractions may aggravate the penetration of the IUD.^[13]^ Risk factors that impact the occurrence of uterine perforation include breastfeeding and postpartum state, experience of the inserting doctor and uterine anatomy.^[5,14–16]^ Women who were breastfeeding at the time of insertion have a 6-fold higher perforation risk than women who were not breastfeeding.^[5]^ Malpositioned IUDs may lead to a high rate of pregnancy due to the possible reduced efficacy.^[17]^ In our case, the patient became pregnant 3 months after IUD insertion, which may have resulted from malposition of the IUD before pregnancy. Studies showed that the type of IUD did not affect the risk of uterine perforation, although stainless steel IUDs were less effective for contraception and easier to expulse.^[5,14]^ As the incidence of IUD malposition is increased in patients with uterine structure abnormality,^[16]^ it was not clear whether the partial septate uterus in this patient contributed to the IUD translocation.

Eighty-five percent of perforations do not affect other organs, and omentum or bowel adhesion formation is the most common complication.^[13]^ Rarely, IUDs protrude into the adjacent viscera, especially the intestinal tract, such as appendix and rectum.^[13]^ Other unusual sites of IUD migration have been reported, including bladder, ureter, and inguinal region.^[8,18,19]^ Patients can be asymptomatic or have serious complications, such as fistulas, intra-abdominal abscesses, intestinal perforations or renal failure.^[13,18,20,21]^

Various imaging modalities are useful in the evaluation of complications of IUD. US is the first choice for the initial evaluation because of its convenience, low cost, and lack of radiation. The US findings of IUDs are echogenic structures with acoustic shadows. It is notable that not all IUDs can be visualized on US. Levonorgestrel-releasing intrauterine systems are more challenging to visualize than copper IUDs due to the use of barium sulfate, which is radiographic but not sonographic.^[22]^ Plain radiography can be helpful in detecting extrauterine IUDs. CT is the best modality to evaluate the location of the IUD and the complications associated with perforation, such as bowel obstruction and abscess formation. Moreover, other diseases can be ruled out on CT scans when patients are symptomatic. Magnetic resonance imaging (MRI) is not routinely used to evaluate IUDs, although modern IUDs are safe for MRI and are mainly made of copper and silver. However, this is not the case for stainless steel ring IUDs, which has been the main type of IUD advocated for by the National Family Planning Program in China since the 1950 s.^[23]^ Bussmann et al^[3]^ found that the stainless steel ring not only produced prominent artifacts but also experienced remarkable dislocation on 1.5 and 3.0 T MRI, indicating that these IUDs are unsafe for MR examinations.

The management of intraperitoneal IUDs is controversial. Some researchers have suggested that surgical intervention is not necessary, as there may be no adhesion formation related to IUD migration in asymptomatic patients.^[24,25]^ However, many researchers hold the opposite view that removal of the intraperitoneal IUD can prevent possible complications, even if patients are asymptomatic.^[9,14,26]^

It is extremely rare for IUDs to migrate into ovarian tumors. In 2008, Koo et al^[27]^ reported the first case that in which an IUD migrated into an ovarian serous adenocarcinoma 36 years after IUD insertion. Ours was the second reported case to describe this issue. The mechanism of IUD migration to the ovarian tumor is unclear. It may be that the IUD initially perforated the normal ovary before the development of the tumor or that the IUD penetrated an ovarian tumor that already existed. To date, no studies have illuminated the correlation between IUD migration into the ovary and the development of ovarian tumors. Theoretically, inflammatory reactions resulting from chronic irritation caused by migrated IUDs may promote tumorigenesis,^[28]^ although a large-scale prospective cohort study from the Shanghai Women's Health Study found that using IUDs (mainly stainless steel rings) for more than 20 years could reduce the risk for ovarian cancer compared to that in never-users (hazard ratio: 0.62, 95% confidence interval: 0.40–0.97).^[29]^ Further studies are needed to investigate the possible relationship between IUD translocation and ovarian cancer tumorigenesis, which may affect the management of extrauterine displaced IUDs.

In conclusion, migration of an IUD into an ovarian tumor is extremely rare. This is the second report in the English literature describing an extrauterine IUD within an ovarian tumor. The correlation between ovarian cancer tumorigenesis and IUD translocation requires further investigation.

## Author contributions

**Conceptualization:** Yongyu An.

**Investigation:** Chang Liu.

**Supervision:** Guangzhao Yang.

**Validation:** Guoqun Mao.

**Visualization:** Fan Mao.

**Writing – original draft:** Yongyu An.

**Writing – review & editing:** Guoqun Mao.

## References

[1] Kavanaugh ML Jerman J Finer LB Changes in use of long-acting reversible contraceptive methods among U.S. women, 2009-2012. Obstet Gynecol 2015;126:917–27. 26444110 10.1097/AOG.0000000000001094 PMC4946164

[2] Arrowsmith ME Majeed A Lee JT Impact of pay for performance on prescribing of long-acting reversible contraception in primary care: an interrupted time series study. PLoS One 2014;9:e92205. 24694949 10.1371/journal.pone.0092205 PMC3973652

[3] Bussmann S Luechinger R Froehlich JM Safety of intrauterine devices in MRI. PLoS One 2018;13:e204220. 10.1371/journal.pone.0204220 PMC6177157 30300364

[4] Kaislasuo J Suhonen S Gissler M Intrauterine contraception: incidence and factors associated with uterine perforation-a population-based study. Hum Reprod 2012;27:2658–63. 22763376 10.1093/humrep/des246

[5] Heinemann K Reed S Moehner S Risk of uterine perforation with levonorgestrel-releasing and copper intrauterine devices in the European Active Surveillance Study on Intrauterine Devices. Contraception 2015;91:274–9. 25601352 10.1016/j.contraception.2015.01.007

[6] Harrison-Woolrych M Ashton J Coulter D Uterine perforation on intrauterine device insertion: is the incidence higher than previously reported? Contraception 2003;67:53–6. 12521659 10.1016/s0010-7824(02)00417-1

[7] Van Houdenhoven K van Kaam KJ van Grootheest AC Uterine perforation in women using a levonorgestrel-releasing intrauterine system. Contraception 2006;73:257–60. 16472566 10.1016/j.contraception.2005.08.013

[8] Chai W Zhang W Jia G Vesical transmigration of an intrauterine contraceptive device: A rare case report and literature review. Medicine (Baltimore) 2017;96:e8236. 28984781 10.1097/MD.0000000000008236 PMC5738017

[9] Kho KA Chamsy DJ Perforated intraperitoneal intrauterine contraceptive devices: diagnosis, management, and clinical outcomes. J Minim Invasive Gynecol 2014;21:596–601. 24462588 10.1016/j.jmig.2013.12.123 PMC6661232

[10] Uysal G Nazik H Tanridan ON Surgical removal of an extrauterine device migrating to appendix. Case Rep Med 2016;2016:4732153. 27885327 10.1155/2016/4732153 PMC5112331

[11] Jatlaoui TC Riley H Curtis KM The safety of intrauterine devices among young women: a systematic review. Contraception 2017;95:17–39. 27771475 10.1016/j.contraception.2016.10.006 PMC6511984

[12] Stephen SE The intrauterine device and the intrauterine system. Best Pract Res Clin Obstet Gynaecol 2014;28:807–24. 24947600 10.1016/j.bpobgyn.2014.05.004

[13] Zakin D Stern WZ Rosenblatt R Complete and partial uterine perforation and embedding following insertion of intrauterine devices. I. Classification, complications, mechanism, incidence, and missing string. Obstet Gynecol Surv 1981;36:335–53. 7029368 10.1097/00006254-198107000-00001

[14] Sun X Xue M Deng X Clinical characteristic and intraoperative findings of uterine perforation patients in using of intrauterine devices (IUDs). Gynecol Surg 2018;15:3–9. 29386988 10.1186/s10397-017-1032-2 PMC5770510

[15] Heinemann K Barnett C Reed S IUD use among parous women and risk of uterine perforation: a secondary analysis. Contraception 2017;95:605–7. 28322770 10.1016/j.contraception.2017.03.007

[16] Gerkowicz SA Fiorentino DG Kovacs AP Uterine structural abnormality and intrauterine device malposition: analysis of ultrasonographic and demographic variables of 517 patients. Am J Obstet Gynecol 2019;220:181–3. 30419198 10.1016/j.ajog.2018.11.122

[17] Braaten KP Benson CB Maurer R Malpositioned intrauterine contraceptive devices: risk factors, outcomes, and future pregnancies. Obstet Gynecol 2011;118:1014–20. 22015868 10.1097/AOG.0b013e3182316308

[18] Li X Li H Li C Migration of an intrauterine device causing severe hydronephrosis progressing to renal failure: a case report. Medicine (Baltimore) 2019;98:e13872. 30653092 10.1097/MD.0000000000013872 PMC6370023

[19] Aghaways I Anwer WS Ali R Migration of an intrauterine device to the left inguinal region, the first reported case. Int J Surg Case Rep 2016;28:68–70. 27689521 10.1016/j.ijscr.2016.09.030 PMC5043400

[20] Pirwany IR Boddy K Colocolic fistula caused by a previously inserted intrauterine device. Case report. Contraception 1997;56:337–9. 9437564 10.1016/s0010-7824(97)00161-3

[21] Ozgun MT Batukan C Serin IS Surgical management of intra-abdominal mislocated intrauterine devices. Contraception 2007;75:96–100. 17241837 10.1016/j.contraception.2006.09.011

[22] Moschos E Twickler DM Does the type of intrauterine device affect conspicuity on 2D and 3D ultrasound? AJR Am J Roentgenol 2011;196:1439–43. 21606311 10.2214/AJR.10.5483

[23] Bilian X Chinese experience with intrauterine devices. Contraception 2007;75: 6 Suppl: S31–4. 17531613 10.1016/j.contraception.2006.12.007

[24] Markovitch O Klein Z Gidoni Y Extrauterine mislocated IUD: is surgical removal mandatory? Contraception 2002;66:105–8. 12204783 10.1016/s0010-7824(02)00327-x

[25] Adoni A Ben CA The management of intrauterine devices following uterine perforation. Contraception 1991;43:77–81. 1825971 10.1016/0010-7824(91)90128-3

[26] Balci O Mahmoud AS Capar M Diagnosis and management of intra-abdominal, mislocated intrauterine devices. Arch Gynecol Obstet 2010;281:1019–22. 20157719 10.1007/s00404-010-1374-8

[27] Koo HR Oh YT Kim YT Intrauterine device found in an ovarian carcinoma. J Comput Assist Tomogr 2008;32:69–71. 18303291 10.1097/RCT.0b013e31805b7ed9

[28] Macciò A Madeddu C Inflammation and ovarian cancer. Cytokine 2012;58:133–47. 22349527 10.1016/j.cyto.2012.01.015

[29] Huang Z Gao Y Wen W Contraceptive methods and ovarian cancer risk among Chinese women: a report from the Shanghai women's health study. Int J Cancer 2015;137:607–14. 25556333 10.1002/ijc.29412 PMC4437849

